# The Neuroscience Peer Review Consortium

**DOI:** 10.1111/j.1460-9568.2009.06643.x

**Published:** 2009-02

**Authors:** Clifford B Saper, John HR Maunsell, On behalf of the NPRC Editors and Publishers

**Affiliations:** E-mail: csaper@bidmc.harvard.edumaunsell@hms.harvard.edu

As the Neuroscience Peer Review Consortium (NPRC) ends its first year, it is worth looking back to see how the experiment has worked.

NPRC was conceived in the summer of 2007 at a meeting of editors and publishers of neuroscience journals. One of the working groups addressed whether it was possible to construct a system for permitting authors whose manuscript received supportive reviews at one journal but was not accepted (perhaps because it was not within the scope of the first journal, or not sufficiently novel to merit publication in a general journal and therefore better for a speciality journal) to send a revised manuscript together with its first round of reviews to a new journal for the second round. This would speed up the review process and reduce the work for reviewers and editors.

The working group not only designed a framework for transferring reviews among journals, but also implemented it as the NPRC. By the autumn of 2007, more than a dozen major journals had signed onto the NPRC, sufficient to launch the experiment in January, 2008. As of the autumn of 2008, 33 journals belong to the Consortium (Table 1). For details about the NPRC, you can go to its website at http://nprc.incf.org. You will find information for Authors, Reviewers, Editors, and Publishers there, as well as the information on how journals can join the Consortium.

**Table 1:** The NPRC as of November 19, 2008

Journals in the NPRC:
Behavioral and Brain Functions
Behavioral Neuroscience
Biological Psychiatry
Brain Research
Brain Structure and Function
CNS Spectrums
Developmental Neuroscience
European Journal of Neuroscience
European Psychiatry
Experimental Neurology
Hippocampus
Human Brain Mapping
Journal of Alzheimer’s Disease
Journal of Comparative Neurology
Journal of Computational Neuroscience
Journal of Integrative Neuroscience
Journal of Neurophysiology
Journal of Neuroscience
Journal of the Association for Research in Otolaryngology
Learning and Memory
Molecular and Cellular Neuroscience
Nature Neuroscience
Neural Development
Neural Plasticity
Neurobiology of Disease
Neurobiology of Learning and Memory
Neuroendocrinology
NeuroImage
Neuroinformatics
Neuropharmacology
Neuroscience
Neuroscience Letters
Psychophysiology
Journals in the process of joining the NPRC:
Cerebrovascular Diseases
Journal of Neuroinflammation
Journal of Neuropathology and Experimental Neurology
Neurobiology of Aging
Restorative Neurology and Neuroscience

The editors of Consortium journals were recently polled to determine how the NPRC has been working. They responded that during the first 9 months about 1–2% of manuscripts that they received had been forwarded from another Consortium journal. A similar number had been sent out from each journal to other participants. In most cases, the papers had been expedited, because the editors at the second journal felt the previous reviews, and the authors’ response to them, were sufficiently positive to permit re-review by one or both of the original referees. In those cases when the editor at the second journal felt that they needed to get new reviews, the review time at the second journal was about what it would have been if the paper had been submitted there by ordinary means.

So, the savings in time and labor are considerable for most of the papers that are transferred between journals via the NPRC. Why then are so few authors using this option?

## Broadening the net

One reason may be that authors resubmit their manuscripts to a journal outside the NPRC. The Consortium includes journals with large volumes of submissions and publications, but the list is far from complete. For example, ISI Web of Knowledge lists 211 Neuroscience journals. The Consortium currently spans this spectrum of journals, from very general to highly specific. However, as more journals join the NPRC, the utility of the system will undoubtedly increase.

A more likely reason for authors not using the NPRC is that they are simply not aware of it. Although there were attempts to publicize the NPRC at its onset, many authors may not know about the possibility, or know which journals participate.

The process of transferring a paper from one journal to another could not be easier. The author simply revises the paper in response to the original reviews, and writes a cover letter that lists the changes that have been made, the name of the journal at which the paper was previously reviewed, and the accession number at the previous journal. When the paper is submitted to the second journal, the author notes the new accession number and then sends an email to the first journal (contact information for editorial offices is on the NPRC website), asking them to send the reviews for their manuscript to the second journal (giving both the accession number at the first journal, and the new accession number at the second journal). The first journal will then send the reviews directly to the second journal, including the names of the reviewers (if they have agreed to have their names transferred). The editors at the second journal then can treat the paper as they see fit, based on the first set of reviews.

Of course, not all papers (and reviews) lend themselves to this process. If the reason for rejection at the first journal is that the referees had substantive requirements for additional work or revisions, authors may decide to revise the paper, but then start fresh at the second journal. In the end, we estimate that it is not likely that more than about 10% of rejected manuscripts are appropriate to be handled via the NPRC. But given rejection rates between 50% and 80% at many of the consortium journals, many papers could benefit from the NPRC, and certainly many more than are currently using it.

## The future of the NPRC

The current members of the NPRC decided in November to extend the life of the Consortium, which was originally a one-year experiment, by at least another year. The International Neuroinformatics Coordinating Facility (INCF), which provides the infrastructure for the NPRC, has agreed to provide its resources for another year. The intention is to continue forward on a year-to-year basis, at the voluntary participation of the member journals. We have in particular to thank Jan Bjaalie, the director of the INCF, and Elli Chatzopoulou, who has been doing all of the administrative work in the INCF, for supporting the NPRC.

We invite authors who have not yet used the NPRC to try this method for appropriate manuscripts. We invite journal editors and publishers who have held back during the first year to join in. The NPRC entails virtually no cost or work, and provides a payoff in reduced work for authors, reviewers and editors. The methods for authors and editors to use the NPRC are clearly outlined in its website (http://nprc.incf.org). Those who have questions are encouraged to contact us by Email.

## Signed

Alain Destexhe and Barry Richmond, Journal of Computational Neuroscience

Anthony Campagnoni, Developmental Neuroscience

Jean-Marc Fritschy and Martin Sarter, European Journal of Neuroscience

Clifford Saper, Journal of Comparative Neurology

Floyd E. Bloom, Brain Research

George Perry and Mark A. Smith, Journal of Alzheimer Disease

John Maunsell, Journal of Neuroscience

Giorgio Ascoli, David Kennedy, and Erik De Schutter, Neuroinformatics

Howard Eichenbaum, Hippocampus

J. Timothy Greenamyre, Neurobiology of Disease

John Krystal, Biological Psychiatry

Karl Zilles and Laszlo Zaborszky, Brain Structure and Function

John H.Byrne, Learning and Memory

Mark Blumberg, Behavioral Neuroscience

Andrew Lumsden, Bill Harris, Joshua Sanes, and Rachel Wong, Neural Development

Ruth Anne Eatock, Journal of the Association for Research in Otolaryngology (JARO)

Sid Gilman, Experimental Neurology

Sophia Frangou, European Psychiatry

Terje Sagvolden, Behavioral and Brain Functions

Ole Petter Ottersen and Stephen Lisberger, Neuroscience

Roman R. Poznanski, Journal of Integrative Neuroscience

Robert F. Simons, Psychophysiology

Robert Millar, Neuroendocrinology

Peter T. Fox and Jack L. Lancaster, Human Brain Mapping

Paul Fletcher, NeuroImage

